# Comparison of efficacy of a 7-day versus a 14-day course of intravenous antibiotics in the treatment of uncomplicated neonatal bacterial sepsis: study protocol of a randomized controlled non-inferiority trial

**DOI:** 10.1186/s13063-021-05785-6

**Published:** 2021-11-29

**Authors:** Sourabh Dutta, Sushma Nangia, Mamta Jajoo, Geeta Gathwala, Saudamini Nesargi, Mangalabharathi Sundaram, Praveen Kumar, Arvind Saili, Dipti Kumar, Poonam Dalal, P. N. Suman Rao, Ramya Shanmugam, Pallab Ray, Valinderjeet Singh Randhawa, Karnika Saigal, Madhu Sharma, Savitha Nagaraj, Devasena Radhakrishnan

**Affiliations:** 1 Neonatology Unit, Department of Pediatrics, Postgraduate Institute of Medical Education and Research, Chandigarh, India; 2 Lady Hardinge Medical College & Kalawati Saran Children’s Hospital, New Delhi, India; 3 Chacha Nehru Bal Chikitsalaya, New Delhi, India; 4 Postgraduate Institute of Medical Sciences, Rohtak, India; 5 St John’s Medical College and Hospital, Bengaluru, India; 6 Institute of Child Health, Madras Medical College, Chennai, India

**Keywords:** Neonate, Sepsis, Blood culture, Antibiotics, Duration, Randomized controlled trial

## Abstract

**Background:**

Neonatal sepsis is a global public health problem. There is no consensus regarding the optimum duration of antibiotics for culture-proven neonatal sepsis. Published randomized controlled trials (RCTs) comparing shorter versus longer courses of antibiotics provide low-quality evidence with serious risk of bias. We hypothesized that among neonates with uncomplicated culture-proven sepsis, antibiotic duration of 7 days is not inferior to 14 days.

**Methods:**

This is a multi-centric, parallel-group, stratified, block-randomized, active-controlled, non-inferiority trial with outcome assessment blinded. Stratification is by center and birth weight. Neonates weighing ≥1000 g at birth, with blood-culture-proven sepsis (barring *Staphylococcus aureus* and fungi), without conditions warranting > 14 days antibiotics, and who clinically remit, are enrolled in the RCT on day 7 of administration of sensitive antibiotics. They are randomly allocated to no further antibiotics (intervention arm: total 7 days) or 7 more days of the same antibiotics (control arm: total 14 days). Allocation is concealed by opaque, sealed envelopes. The primary outcome is “definite or probable relapse” within 21 days after antibiotic completion. Secondary outcomes include definite and probable relapses at various timepoints until day 35 post-randomization, secondary infections, and adverse events. The neonatologist adjudicating probable relapses and lab personnel are blinded. Three hundred fifty subjects will be recruited in each arm, assuming a non-inferiority margin of 7%, one-sided alpha error 5%, and power of 90%. Analysis will be per protocol and by intention-to-treat. An independent Data Safety Monitoring Board monitors adverse events and will perform one interim analysis when 50% of expected primary outcomes have occurred or 50% of subjects have completed follow-up, whichever is earlier. O’Brien-Fleming criteria will be used to stop for mid-term benefit and Pocock’s to stop for mid-term harm. A priori subgroup analyses are planned by birth weight categories, gram-stain status of pathogens, and radiological pneumonia.

**Discussion:**

This trial will provide evidence to guide practice regarding optimum duration of antibiotics for culture-proven neonatal bacterial sepsis. If a 7-day regime is proved to be non-inferior to a 14-day regime, it is likely to reduce hospital stay, costs, adverse effects of drugs, and nosocomial infections.

**Trial registration:**

Clinical Trials Registry India CTRI/2017/09/009743. Registered on 13 September 2017.

**Supplementary Information:**

The online version contains supplementary material available at 10.1186/s13063-021-05785-6.

## Administrative information

**Table:** 

Title {1}x	Comparison of the efficacy of a 7-day versus a 14-day course of intravenous antibiotics in the treatment of uncomplicated neonatal bacterial sepsis: a randomized controlled non-inferiority trial
Trial registration {2a and 2b}.	Clinical Trials Registry-India (CTRI): registration number CTRI/2017/09/009743 ClinicalTrials.gov: ID NCT03280147.
Protocol version {3}	Version 3, 5 February 2019
Funding {4}	Funded by Indian Council of Medical Research (ICMR), Ansari Nagar, New Delhi, India
Author details {5a}	Sourabh Dutta^a^, Sushma Nangia^b^, Mamta Jajoo^c^, Geeta Gathwala^d^, Saudamini Nesargi^e^, Mangalabharathi Sundaram^f^, Praveen Kumar^a^, Arvind Saili^b^, Dipti Kumar^c^, Poonam Dalal^d^, Suman Rao P. N.^e^, Ramya Shanmugam^f^, Pallab Ray^a^, Valinderjeet Singh Randhawa^b^, Karnika Saigal^c^, Madhu Sharma^d^, Savitha Nagaraj^e^, Devasena Radhakrishnan^f^. ^a^ Postgraduate Institute of Medical Education and Research, Chandigarh, India ^b^ Lady Hardinge Medical College & Kalawati Saran Children’s Hospital, New Delhi, India ^c^ Chacha Nehru Bal Chikitsalaya, New Delhi, India ^d^ Postgraduate Institute of Medical Sciences, Rohtak, India ^e^ St John’s Medical College and Hospital, Bengaluru, India ^f^ Institute of Child Health, Madras Medical College, Chennai
Name and contact information for the trial sponsor {5b}	Director-general, ICMR, V. Ramalingawami Bhawan, Ansari Nagar, Post Box Bo. 4911 New Delhi - 110029
Role of sponsor {5c}	ICMR will have no role in study design, data collection, data management, analysis and interpretation of data, writing of the report or decision to submit the report for publication.

## Introduction

### Background and rationale {6a}

Neonatal sepsis is an important cause of morbidity and mortality. Globally, neonatal sepsis accounts for 8% of all neonatal deaths in the 1st week of life and 37% of all deaths from the 2nd to 4th weeks of life [1]. In hospital settings, the incidence of culture-proven neonatal sepsis is 16 per 1000 live births in India [2]. One large study from a rural community in India reported 4 cases of culture-proven neonatal sepsis per 1000 live births [3]. Population-based studies from India report highly variable incidences of clinically suspected sepsis—ranging from 4.6 to 170 per 1000 live births [4]. Given the incidence of sepsis, the use of antibiotics in the neonatal period is high all over the world, particularly so in India. The overuse and prolonged use of antibiotics has resulted in an alarming problem of multidrug-resistant (MDR) neonatal sepsis. In South Asia, most isolates of *Klebsiella pneumoniae*, *Escherichia coli*, and *Acinetobacter baumannii* are MDR [2]. The reliance on newer generations of antibiotics has also increased the cost of care and the incidence of serious adverse events (SAE).

In view of these problems, it is important to optimize the duration of antibiotic therapy. If shorter courses of antibiotics are found to be equally efficacious, without an increased risk of relapses, complications, or mortality, then shorter courses could safely replace longer courses. Shorter courses of antibiotics would be expected to cause less SAEs, require shorter hospitalization, incur less cost, and decrease the risk of secondary bacterial infections. When scaled up to the level of the community, the benefits, if any, of shorter courses of antibiotics would be enormous, resulting in many more hospital beds being freed up, less financial burden on the public health system, less fungal sepsis, and less antimicrobial resistance.

There is no consensus in clinical practice regarding the optimal duration of antibiotic therapy for culture-proven neonatal sepsis. Pediatric and neonatology textbooks mention figures between 1 week and 2 weeks of therapy, with most units prescribing 10–14 days of antibiotics for culture-proven uncomplicated neonatal septicemia.

We performed an extensive literature search of PubMed, Embase, and the Cochrane Database Of Systematic Reviews from January 1990 to June 2020 to identify clinical trials that compared a short course versus a standard course of antibiotics for the treatment of uncomplicated culture-proven neonatal bacterial septicemia. We reviewed 2453 titles and abstracts. Based on the abstracts, we reviewed 6 full-text articles. We did not find any eligible meta-analyses. We found only 3 small randomized controlled trials (RCTs) on this research question [5–7]. We performed our own meta-analysis of these 3 RCTs (unpublished).

Two RCTs reported on the duration of hospitalization [6, 7]. Quality of evidence was low because of a serious risk of bias. The absolute effect ranged from 2.85 to 4.4 fewer days of hospitalization with a shorter course of antibiotics. Although no RCTs reported on the actual duration of antibiotic therapy, it can be surmised that subjects in the short course antibiotics arms would have received fewer days of antibiotics. There were no RCTs addressing death before hospital discharge, and long-term death or neurodevelopmental impairment. Three RCTs reported on mortality by day 28 of life [5–7]. Quality of evidence was very low because of serious risk of bias and very serious imprecision. The absolute effect on mortality by day 28 of life ranged from 33 fewer deaths to 229 more deaths per 1000 subjects.

All 3 RCTs reported on relapse with culture-positive sepsis or meningitis, and relapse with suspected (culture-negative) sepsis or meningitis [5–7]. Quality of evidence was very low because of serious risk of bias and very serious imprecision. The absolute effect on relapse with culture-positive sepsis or meningitis ranged from 20 fewer to 50 more culture-positive relapses per 1000 subjects. The absolute effect on probable relapse with culture-negative sepsis or meningitis ranged from 14 fewer to 137 more relapses per 1000 subjects.

None of the previously performed RCTs were designed as non-inferiority trials. In view of the paucity of information in the published literature, we planned to conduct an adequately powered non-inferiority trial.

### Objectives {7}

We hypothesized that among newborn infants with uncomplicated culture-proven neonatal sepsis who are treated with intravenous antibiotics and who clinically remit by 7 days of appropriate antibiotics, discontinuation of antibiotics (i.e. a total of 7 days antibiotics) is not inferior compared to continuation of antibiotics for another week (i.e. a total of 14 days antibiotics) with respect to definite or probable relapse of sepsis within a 21-day period of observation after antibiotic completion in an RCT.

### Trial design {8}

This is a multi-center, parallel-group, randomized, active-controlled, non-inferiority trial with outcome assessment blinded. Stratified, block randomization is performed. The study population is stratified as per the center and birth weight (> 1000–1500 g, 1501–2000 g, and > 2000 g), and with permuted even-numbered, randomly varying block sizes.

## Methods: Participants, interventions, and outcomes

### Study setting {9}

The study was started in 6 centers across India. These are Postgraduate Institute of Medical Education and Research, Chandigarh; Lady Hardinge Medical College & Kalawati Saran Children’s Hospital, New Delhi; Chacha Nehru Bal Chikitsalaya, New Delhi; Postgraduate Institute of Medical Sciences, Rohtak; St John’s Medical College and Hospital, Bengaluru; and Institute of Child Health, Madras Medical College, Chennai.

### Eligibility criteria {10}

All neonates weighing ≥ 1000 g at birth, with clinically suspected sepsis for which the treating physician decides to start antibiotics are eligible for screening. Neonates who meet the eligibility criteria (mentioned below) are enrolled in the observational part of the study. They are observed until they have received 7 days of sensitive antibiotics for blood culture-proven sepsis, if any. They are re-evaluated on the seventh day to assess whether they meet the eligibility criteria for the RCT part of the study.

#### Eligibility criteria for the observational part of the study

##### Inclusion criteria

Subjects should satisfy *all* the following criteria:

- Neonates aged 0–28 days, who are currently admitted in the neonatal unit of the center,
- Birth weight ≥1000 g,
- Residence is within approximately 15 km from the center, so that the infant can be brought back to the center for follow-up.
- Suspected septicemia for which a conventional or BACTEC/BACT-ALERT blood culture is sent and for which the treating physician decides to start antibiotics.

##### Exclusion criteria (any one of the following)

- Central Nervous System infection [meningitis will be defined as one or more of: cerebrospinal fluid (CSF) cell count ≥ 25 per microliter with > 60% neutrophils; glucose < 20 mg/dl or CSF: blood glucose < 0.6 or protein > 150 mg/dL in term or > 180 mg/dL in preterm or positive gram stain report or positive culture report
- Septic arthritis, osteomyelitis, or deep-seated abscess as clinically judged by the treating team
- Life-threatening congenital malformations as judged by the principal investigator of the center.

Parents of the subjects who satisfy the eligibility criteria are approached for participation in the study. They are provided a Patient Information Sheet (written in non-technical language). The design and purpose of the observational part of the study is discussed with them and they are informed that they may be approached again for the RCT portion of the study after approximately 7 days if the infant meets the criteria for randomization. Written informed consent for enrollment is taken from a parent in the presence of a witness.

#### Data of observational part of the study

After obtaining consent for enrollment, the following data is recorded: maternal and neonatal demographic data, maternal risk factors of sepsis, clinical and laboratory features of sepsis, culture, and sepsis workup reports, antibiotics, and their doses. The subjects are followed up daily for the remission of clinical signs (using a standard objectively defined list), change of antibiotics (if any, with reasons), and co-interventions. Each participating center decides on the empirical antibiotics based upon its standard empirical antibiotic policy. The antibiotics are stored, prescribed, and dispensed as per the recommendations of Neofax essentials, 2014 [8]. All study subjects receive standard care as per the guidelines of the concerned center.

#### Randomization criteria

Randomization is done at the end of the 7th day of therapy with sensitive antibiotics. Only those who satisfy the criteria below are randomized:

##### Eligible for randomization

1. Positive blood culture
2. No signs and symptoms of sepsis from the end of day 5 through to the end of day 7 of starting sensitive antibiotics

##### Not eligible for randomization

- Suspected contaminants in blood culture.
- Growth of *Staphylococcus aureus* in blood culture
- Growth of fungal organism in blood culture
- Diagnosis of meningitis, septic arthritis, osteomyelitis, abscess
- Ambiguity regarding in vivo sensitivity of antibiotics used

### Who will take informed consent? {26a}

The principal investigator at each participating center obtains informed consent.

### Additional consent provisions for collection and use of participant data and biological specimens {26b}

There is no provision for additional consent for collection and use of participant data and biological specimens, as it is not relevant to this RCT.

## Interventions

### Explanation for the choice of comparators {6b}

Participants eligible for the RCT are randomly allocated to receive either a total of 7 days of appropriate antibiotics or a total of 14 days of appropriate antibiotics. Here, 14 days is assumed to be the standard course of antibiotics for culture-proven neonatal bacterial sepsis, and 7 days is assumed to be the intervention (a short course of antibiotics).

### Intervention description {11a}

Participants in the intervention arm do not receive any more antibiotics after randomization. They would have already received 7 days of appropriate antibiotics, to which the etiologic organism is sensitive. The control arm receives 7 more days of appropriate antibiotics. Thus, participants in the control arm would receive a total of 14 days of appropriate antibiotics, 7 days of which would have already been received before randomization and 7 days after randomization. The choice of initial antibiotics would be according to the antibiotic sensitivity pattern of the participating center and would be left to the discretion of the clinical team.

All other co-interventions and supportive care would be provided according to the local unit protocols and would be identical for both the arms of the study.

### Criteria for discontinuing or modifying allocated interventions {11b}

If a participant enrolled in the RCT worsens and is suspected to have a fresh episode of sepsis, a blood culture, and other sepsis workup is performed, and new antibiotics are either started (in the 7-day arm) or upgraded (in the 14-day arm). The choice of empiric antibiotics for a fresh episode of sepsis are as per the empiric antibiotic policy of the unit.

### Strategies to improve adherence to interventions {11c}

As all subjects receive intravenous antibiotics administered by nursing staff, compliance of participants to the interventions is not expected to be an issue. If a subject in the 14-day arm is referred from the center to a stepdown care unit, the research staff at the center communicates the exact antibiotic protocol to the staff of the stepdown care unit and telephonically monitor adherence to the intervention daily.

### Relevant concomitant care permitted or prohibited during the trial {11d}

There is no concomitant care that is specifically permitted or prohibited during the trial.

### Provisions for post-trial care {30}

In the RCT part of the study, participants are regularly monitored for the appearance of adverse events (AE). Details regarding the notification of AEs are mentioned elsewhere in this manuscript. Participants in the trial are insured under a clinical trials insurance. Deaths and SAE attributed to participation in the trial are compensated as per government regulations [9]. Treatment of any AEs arising from participation in the trial is provided free of cost.

### Outcomes {12}

#### Primary outcome variable

“Definite or probable relapse”. Definite relapse is defined as the occurrence of an episode of illness within the 21-day period after antibiotic completion in which the same organism with similar antibiogram is grown, as in the original episode. Probable relapse is defined as the occurrence of an episode of illness within the 21-day period, when the episode is diagnosed to be a relapse of bacterial sepsis based on clinical features and investigations, in the setting of a sterile blood culture. Probable relapse is adjudicated by a blinded Neonatologist who is not associated with the rest of the study.

Since this is a non-inferiority trial, the analysis will be done both as per protocol and as per intention to treat. Both the analyses will be reported, bearing in mind that the per protocol analysis is the more conservative of the two, with less bias towards “no effect.”

In the per protocol analysis, the following subjects will be excluded from analysis:

- In the 14-day group: patients whose 8th–14th day of the original antibiotics could not be completed for any reason (including, but not limited to non-availability of cannula, a fresh episode of sepsis requiring change of antibiotics, withdrawal of consent or unscheduled discharge),
- Subjects in either group whose primary outcome could not be assessed due to loss to follow-up or withdrawal of consent.

In the intention-to-treat analysis, all randomized subjects will be analyzed according to the group to which they were randomized. A sensitivity analysis (worst-case scenario) will be performed to impute outcomes among subjects who are permanently lost to follow-up.

#### Secondary outcomes

Episodes of relapses (# 1–4) will be analyzed both as per protocol and as per intention-to-treat:

1. “Definite relapse”: by 21 days after antibiotic completion.
2. “Probable relapse”: by 21 days after antibiotic completion.
3. “Definite relapse”: by 28 days after antibiotic completion.
4. “Probable relapse”: by 28 days after antibiotic completion.

Episodes of relapses (# 5-7) will be analyzed as per intention-to-treat:

5. “Definite or probable relapse” in the 28-day period after randomization and 35-day period after randomization
6. “Definite relapse” in the 28-day period after randomization and 35-day period after randomization
7. “Probable relapse” in the 28-day period after randomization and 35-day period after randomization
8. Episodes of proven secondary infections (either bacterial or fungal) during the 35-day period after randomization.
9. AEs: All AEs occurring after randomization are recorded. AEs with onset prior to randomization are recorded only if there is worsening after randomization. AEs are categorized as mild, moderate, severe, life-threatening and death. Severe, life-threatening and death are reported as SAEs, irrespective of whether they are considered related to the interventions in the trial.

### Participant timeline {13}

See Fig. 1

**Fig. 1:**
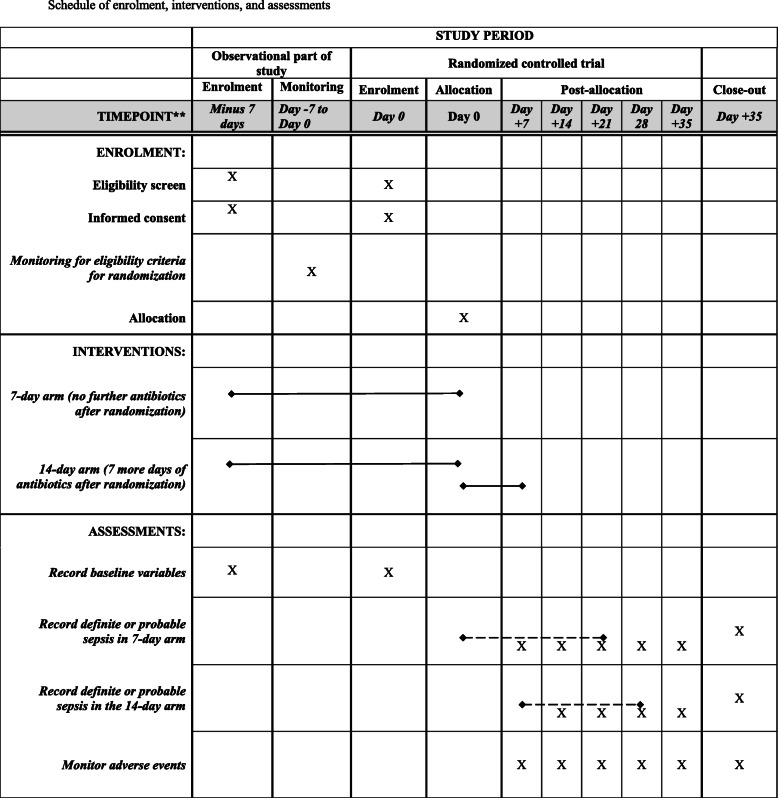
Schedule of enrolment, interventions, and assessments

### Sample size {14}

The sample size is 350 in each arm, assuming event rate for the composite primary outcome (definite or probable relapse) to be 10% based on an earlier study [5], non-inferiority margin of 7%, one-sided alpha of 5%, power of 90%, and lost to follow-up of approximately 10%.

### Recruitment {15}

Whenever any newborn infant aged to 0-28 days is clinically suspected to have sepsis and is started on intravenous antibiotics after sending a blood culture sample, the research team is informed. After obtaining consent for the observational part of the study, the research team tracks the blood culture and sensitivity report of each participant and monitors the participants daily for randomization criteria. If a participant meets the criteria for enrolment in the RCT, the research team approaches the parents, provides them the information sheet for the RCT and seeks written informed consent. Those who do not meet randomization criteria and/or where consent is not available, are not randomized. They are administered the standard 14-days course of antibiotics.

## Assignment of interventions: allocation

### Sequence generation {16a}

The sequence generation is done centrally. Stratified, block randomization is performed. Randomly varying even-numbered, permuted block sizes are used, and size of the blocks will be concealed until the end of the study. Stratification is by birth weight (1000–1500 g, 1501–2000 g, and > 2000 g) and by center. Random number lists generated from http://randomizer.org are converted into the sequence of allocation.

### Concealment mechanism {16b}

Slips bearing the allocated group are placed in opaque envelopes, which are sealed and numbered serially on the outside. Sealed envelopes made centrally and dispatched to each site. There are separate sets of such envelopes, one for each stratum (i.e., center and birth weight group). The name and identification details of the subject are written on the outside of each envelope and all envelopes are returned to the Principal Investigator in the nodal center.

### Implementation {16c}

A statistician unconnected with the trial has generated the random sequence and prepared the sealed envelopes. The principal investigator of each site enrolls the participants and, with the help of the research staff at each site, implements the interventions. The clinical nurses administer antibiotics under the oversight of the research team.

## Assignment of interventions: Blinding

### Who will be blinded {17a}

The following research team members are blinded: the adjudicator of the probable relapses and the laboratory personnel. The following are unblinded: the clinical investigators, the research staff, nurses, and resident doctors involved in the care of the subjects.

Data related to the assessment of the outcomes are recorded in a separate detachable part (part B) of the case report form (CRF). Part B has no data regarding patient identification and group of randomization, except for a unique form serial number. The form serial number will be used for concatenating the two parts of the CRF later. Part B is sent to the blinded adjudicator. The adjudicator decides whether episodes of illness during follow-up are probable relapses of bacterial sepsis or not. The adjudicator is permitted to seek additional anonymized information from the concerned center to facilitate the diagnosis.

### Procedure for unblinding if needed {17b}

This is an open-label trial. There are no circumstances under which the blinded adjudicator and laboratory personnel will be unblinded.

## Data collection and management

### Plans for assessment and collection of outcomes {18a}

Baseline data has been described earlier in this manuscript. After random allocation, subjects are followed up every week for 5 weeks by a research scientist, who is specifically trained to assess signs of neonatal illness. He/she gets all suspected illnesses confirmed by a Neonatologist, who decides about the investigations for sepsis workup. Personnel performing the investigations are blinded to the group of randomization.

### Plans to promote participant retention and complete follow-up {18b}

Complete addresses and telephone numbers of the patients are recorded before discharge. Any further hospitalization after stoppage of the antibiotic course is at the discretion of the treating unit. All subjects are followed up at 48 h after antibiotic completion (±12 h) for any signs of sepsis. If the baby is discharged, and parents do not bring the baby for follow-up, the research staff visit the home of the patient. At the time of discharge, parents are given follow-up cards with the research staff’s names and contact information.

Parents are asked to call the research staff to report each episode of illness during the follow-up period. Pediatric Emergency staff and Outpatient Department staff are also informed about the follow-up cards and asked to inform the research team in case such a baby reports for any illness.

In addition, parents bring the baby to the follow-up clinic by appointment on a weekly basis (+ 2 days) for 35 days after randomization. At each visit, standard questions are asked by the research scientist to ascertain whether an illness has occurred in the preceding week that required antibiotics prescribed by another hospital. If the parents do not bring the baby back for weekly follow-up, the research staff visit the home of the patient. Details of all episodes of illness during the 35-day follow-up period (whether in hospital or after discharge) are recorded by the research staff.

In case the subject dies at a place other than the center during the follow-up period, the principal investigator and research team contact the family and perform a verbal autopsy. The verbal autopsy form is adapted from the one used by the National Health Mission India [10].

### Data management {19}

Participant data is first entered in paper CRF. Each case report form has a unique form serial number. A website has been created for the trial. The website has fillable online versions of the CRF. The online CRFs have range checks for data values. The principal investigator at each center is provided a username and can create a secure password. The blinded adjudicator does not have access to the trial website.

From the CRF, the data is entered on the website by a medical social worker. Correctness of the data entry of all forms is verified by a research scientist. A data entry operator and the principal investigator at the nodal center of the trial have access to the data entered at all centers. The nodal center performs a random check of 10% CRFs. Any errors in the data are reconciled with the staff of the concerned center, and corrections are made by verifying from the paper CRF and primary sources of data. Data backups are taken once a week.

### Confidentiality {27}

Once the data has been cleaned, the data will be anonymized by removing all patient identifiers, barring the unique form serial number. The hard copy CRFs will be held securely in personal custody by the site investigator and will be sent to the nodal center at the end of the trial. The principal investigator at the nodal center will preserve the hard copies of the CRFs from all centers securely in personal custody for 5 years until the last publication from this study.

### Plans for collection, laboratory evaluation, and storage of biological specimens for genetic or molecular analysis in this trial/future use {33}

No biological samples are stored for genetic or molecular analysis in this trial or for future use. To investigate for definite or probable relapse, routine blood, and body fluid samples for the evaluation of sepsis are obtained for any episode of illness during follow-up. None of the samples are stored long-term.

## Statistical methods

### Statistical methods for primary and secondary outcomes {20a}

The primary and secondary outcomes are all binary. Chi-square test or Fisher’s exact test will be used to test statistical significance. *P* value < 0.05 will be assumed to be statistically significant. The magnitude of the effect of each primary and secondary outcome will be expressed as risk ratio with 95% confidence interval. A multivariable logistic regression analysis will be performed for the primary outcome as the dependent binary variable. The following predictor variables (decided a priori) will be forced into the model: group of randomization, birth weight stratum, whether infected with a gram-positive organism, presence of radiological pneumonia (if the treating team did not order a chest x-ray, it would be assumed no radiological pneumonia), gender and the number of days by which the signs of sepsis had remitted.

### Interim analyses {21b}

An independent Data Safety Monitoring Board (DSMB) monitors SAEs in the trial and will perform one interim analysis. The timing of this analysis will be when 50% of the expected primary outcomes have occurred or when 50% of subjects have completed their follow-up as per protocol, whichever is earlier. At the time of interim analysis, the DSMB will revisit the sample size of the study. O’Brien Fleming stopping criteria will be used for the primary outcome while Pocock’s stopping rule for the SAEs. If treatment failure rates are found significantly higher in the 7-day group in mid-term analysis, the trial will be stopped. If a death occurs due to bacteriologically confirmed relapse from the same organism during the follow-up, the allocation group of that subject will be immediately ascertained. If it is the 7-day group, the trial will be stopped. The final decision, if any, to stop the trial prematurely will test entirely with the independent DSMB.

### Methods for additional analyses (e.g., subgroup analyses) {20b}

Sub-group analysis of the primary outcome has been planned for the following sub-groups: stratum-wise, those infected with gram-positive organisms versus gram-negative organisms, and those with radiological pneumonia versus those without radiological pneumonia. For the sub-group analyses, the level of significance will be kept as *p* < 0.01. The *P* value of the test of interaction (Breslow Day’s test) will be used to determine subgroup interaction.

### Methods in analysis to handle protocol non-adherence and any statistical methods to handle missing data {20c}

As it is a non-inferiority trial, an analysis will be performed both per protocol and by intention-to-treat. The per protocol analysis will be considered the primary analysis. Criteria to qualify for the per protocol analysis have been mentioned earlier in this manuscript. Missing data will be imputed by a multiple imputation method.

### Plans to give access to the full protocol, participant level-data and statistical code {31c}

Public access will be provided to the full protocol by way of this manuscript and the protocol that has been uploaded in clinical trials registries. Participant level data and statistical codes will be provided to research investigators on request, if they plan to perform an individual patient data meta-analysis.

## Oversight and monitoring

### Composition of the coordinating center and trial steering committee {5d}

The coordinating center comprises of the nodal clinical principal investigator, the nodal clinical co-investigator, and the nodal microbiology co-investigator. The project review group (i.e., the trial steering committee) comprises of a senior Neonatologist (chairperson), three senior Pediatricians, and one statistician (member), all of whom have long track records in clinical trials. The members of the trial steering committee do not belong to any of the centers involved in the trial and are at arm’s length from the investigators.

The endpoint adjudicator is a senior Neonatologist, who does not belong to any of the participating centers in the trial, and who is at an arms’ length from the rest of the trial.

### Composition of the data monitoring committee, its role and reporting structure {21a}

The DSMB comprises of a senior neonatologist (chairperson), one mid-career Neonatologist, one bioethicist, and one statistician, all of whom have long track records in clinical trials. All members are at arms’ length from the trial. They are independent from the sponsor and the institutions involved in the trial. The mandate of the DSMB is as follows: (a) to perform an interim analysis [details of which are mentioned in the section on interim analysis], (b) to recalculate the sample size of the study after the interim analysis, (c) to receive quarterly reports from each site as mentioned below, and (d) to receive a concurrent copy of the information sent to the SMC regarding every death in the trial.

The nodal center reports the following information every 3 months stratified site-wise for the entire cohort: number screened for observational part of study, pre-enrolment exclusions for observational part, post enrolment exclusion for observational part, number of participants that completed observational part, number ongoing in observational part, number of participants screened for the RCT part of study, pre-enrolment exclusions from the RCT, post enrolment exclusion from the RCT, number that completed the RCT, number ongoing in the RCT, number died during follow-up in the RCT, SAEs among subjects who died (irrespective of whether death was attributed to SAE), SAEs among subjects who survived, number of definite relapses and probable relapses in 21 days and 28 days after antibiotic completion. The DSMB also receives a copy of the SAE reporting form.

The DSMB will perform one interim analysis, details of which have been mentioned earlier. The DSMB will also monitor all deaths in the trial. The DSMB will have the authority to terminate the trial prematurely under conditions that have already been mentioned earlier in this manuscript. The DSMB is independent of the sponsor. None of the members of the DSMB have any conflict of interest to declare.

### Adverse event reporting and harms {22}

An AE log is maintained for all participants in the RCT. The log includes start and end date, severity, outcome, and action taken. A detailed AE chart with definitions of the AE and criteria for classifying the level of severity has been made. These have been adapted from the Common Terminology Criteria for Adverse Events (CTCAE) version 4.03 [11].

All SAEs are notified in a brief format by email to the SMC as soon as possible within 24 h of onset of the SAE. Detailed reports of the SAE are sent to the SMC within 2 days of onset, at 10 days after onset and at resolution, in the form of a hard copy filed in Appendix 1 of schedule Y as prescribed by the Central Drugs Standard Control Organization (CDSCO), Ministry of Health and Family Welfare, Government Of India (9). The SMC at each center is independent of the sponsors, investigators, trial steering committee, and the DSMB. The SMC decides whether the SAE is attributable to participation in the RCT, whether financial compensation must be provided to the family, and whether the treatment of the SAE must be provided free of cost. The SMC uses the World Health Organization-Uppsala Monitoring Centre (WHO-UMC) causality assessment scale for guidance [12].

### Frequency and plans for auditing trial conduct {23}

A trial coordinator visits each center at least once in 6 months and audits the conduct of the trial. He/she examines all the hard copy forms in the binders of each enrolled patient and randomly verifies the entries of at least 15% of the binders with the primary source. This plan is currently shelved because of the COVID-19 pandemic.

### Plans for communicating important protocol amendments to relevant parties (e.g., trial participants, ethical committees) {25}

Important protocol modifications related to eligibility criteria, interventions, co-interventions, outcomes, follow-up, and analysis are communicated to all the investigators, sponsor, institutional ethics committees, future trial participants, trial registries, and the insurance company.

### Dissemination plans {31a}

The trial results will be communicated via conference presentations, journal articles, professional organizations, and clinical practice guideline groups.

## Discussion

Since this has been planned as a non-inferiority trial, the eligibility criteria are stringent. Therefore, identifying eligible participants is expected to be challenging. The trial steering committee will periodically assess recruitment in the RCT and decided upon further continuation depending upon the feasibility of successfully completing the trial.

## Trial status

Current protocol version 3 dated 5 February 2019. Recruitment began on 1 January 2019. The approximate date when recruitment will be completed is 31 January 2022.

## Supplementary Information

**Additional file 1.** Informed consent

## Abbreviations

AE: Adverse event
CDSCO: Central Drugs Standard Control Organization
CRF: Case report form
CSF: Cerebrospinal fluid
CTCAE: Common Terminology Criteria for Adverse Events
CTRI: Clinical Trials Registry-India
ICMR: Indian Council of Medical Research
MDR: Multidrug resistant
RCT: Randomized controlled trial
SAE: Serious adverse event
SMC: Safety Monitoring Committee
WHO-UMC: World Health Organization-Uppsala Monitoring Centre
