# Supplementary figures and images for: Comparison of efficacy of a 7-day versus a 14-day course of intravenous antibiotics in the treatment of uncomplicated neonatal bacterial sepsis: study protocol of a randomized controlled non-inferiority trial
Source: Trials. 2021 Nov 29;22:859. doi: 10.1186/s13063-021-05785-6 (PMC8628047; doi:10.1186/s13063-021-05785-6)

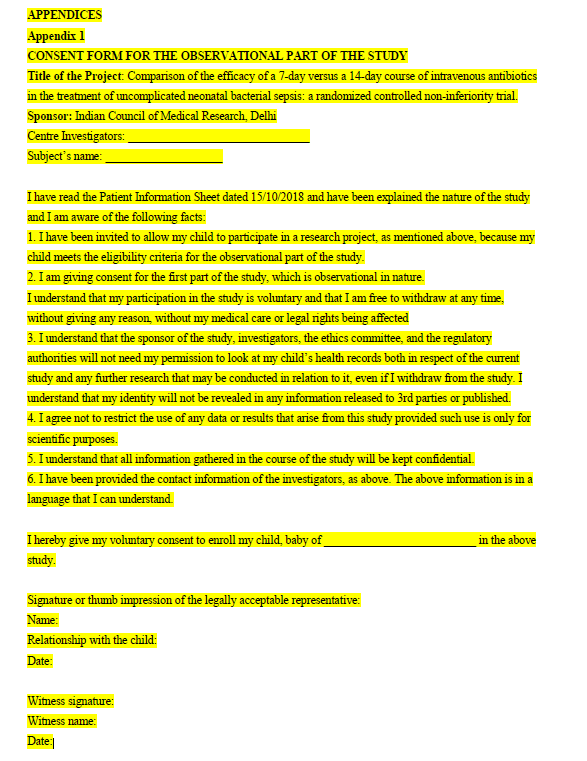


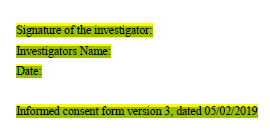


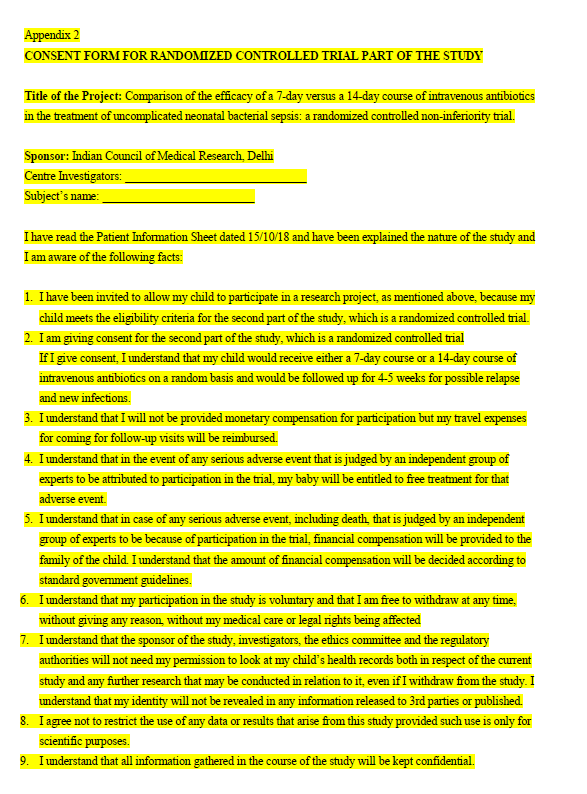


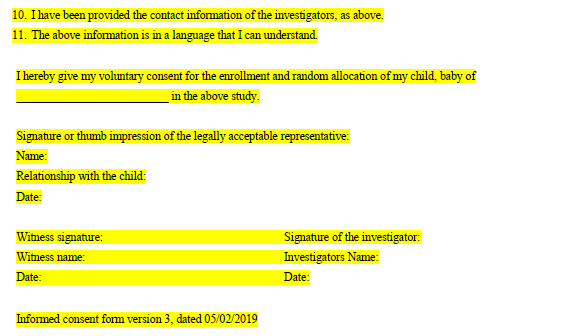

Supplement: Supplementary file 1 — Additional file 1. Informed consent [file 13063_2021_5785_MOESM1_ESM.docx]
